# Managing Multimorbidity (Multiple Chronic Diseases) Amid COVID-19 Pandemic: A Community Based Study From Odisha, India

**DOI:** 10.3389/fpubh.2020.584408

**Published:** 2021-02-01

**Authors:** Sanghamitra Pati, Pranab Mahapatra, Srikanta Kanungo, Azhar Uddin, Krushna Chandra Sahoo

**Affiliations:** ^1^Regional Medical Research Centre, Indian Council of Medical Research, Bhubaneswar, India; ^2^Department of Psychiatry, Kalinga Institute of Medical Sciences, Bhubaneswar, India

**Keywords:** long standing health conditions, comorbidity, SARS-CoV2, MAQ-COVID19, multiple NCD management, health care access, pandemic, multiple morbidities

## Abstract

While most of the studies to date demonstrate the deleterious effect of multiple chronic diseases on COVID-19 risk and outcome, there is sparse information available on the effect of the pandemic on multimorbidity management, with no reports yet from India. We sought to explore the effect of COVID-19 pandemic on routine and emergency care for multimorbidity among community-dwelling adults in Odisha, India. A community-based cross-sectional study was undertaken pandemic lockdown, in Khurda district of Odisha, India. Around 600 individuals having at least one chronic disease residing in rural, urban residential and slums were interviewed using a specifically developed questionnaire MAQ COVID-19. The association of socio-demographic characteristics and multimorbidity with pandemic-related care challenges was examined by multiple logistic regression. Principal Component Analysis was employed to minimize the dimensionality of factors related to multimorbidity care. Multimorbidity was highly prevalent in younger age group (46–60 years) with cardio-metabolic clusters being dominant. Individuals with multimorbidity experienced significantly higher care challenges than those with single condition (AOR = 1.48, 95% CI = 1.01–2.05) with notable disruption in treatment and routine check-up. Most frequently cited concerns were—physician consultation (43%), diagnostic-services (26%), transport (33%), and mobility restrictions (21%). Multivariate analysis revealed older adults living alone in urban residence to have higher challenges than their rural counterparts. Patient activation for self-care, multimorbidity literacy, and technology-enabled tele-consultation could be explored as potential interventions. Future studies should qualitatively explore the challenges of physicians as well as garner an in-depth understanding of multimorbidity management in the vulnerable subgroups.

## Introduction

The emergence and spread of the COVID-19 has resulted in a public health event of widespread concern across the globe in 2020, with the WHO declaring COVID-19 a pandemic in March 2020 (1, 2). According to the available statistics, three countries—the United States of America, Brazil, and India have been most affected with the high absolute number of infected cases (3, 4). India registered the first confirmed case of COVID-19 in January 2020, and more than 8,000 confirmed cases, with almost 200 reported deaths as of May 2020 (5). With an aim to curtail the spread, the nationwide lockdown (1st phase) began from March 24th to May 31st, 2020 in India (6, 7).

Initial epidemiological reports, mostly observational, suggest that individuals with major underlying diseases like chronic kidney disease (CKD), cardiovascular disease (CVD), hypertension (HTN), diabetes, chronic obstructive pulmonary disease (COPD), and malignancy have heightened risk of adverse outcome than their counterparts without any pre-existing disease. Furthermore, recent data reveal that the presence of two or more conditions (multimorbidity) accentuate the outcomes in COVID-19 with around 10-fold risk (8–10).

At the same time, the COVID-19 pandemic has posited an unprecedented stress on healthcare delivery. Various multi-pronged strategies and restrictive measures were adopted by the central and state governments to curb the transmission. Since the onset of COVID-19, health systems have been primarily focusing on pandemic containment and critical care management, which could have impaired routine prevention and curative services including emergency care (11). A rapid global survey conducted by the World Health Organization (WHO) to assess Non-Communicable Diseases (NCDs) management during COVID-19 found NCD services to be impacted, especially in low-and middle-income countries (LMICs) (12). However, the survey focused on single NCD rather than multimorbidity and missing long-standing chronic infections both of which are important issues of concern for LMICs (13). Compared to the western countries, many LMICs are experiencing colliding epidemics of chronic infectious and NCDs leading to increasing prevalence of multimorbidity—the co-occurrence of two or more long-term conditions (14, 15).

While some prior research in India has described the management of NCDs in vulnerable groups during the pandemic (16–18), the same for people having multimorbidity are still missing. Nonetheless, a detailed understanding of multimorbidity management during COVID-19 is essential in India, as one-third of people attending healthcare have multimorbidity (19).

We, therefore, undertook the first study to explore the effect of COVID-19 pandemic on management of multimorbidity among individuals having long-standing health condition during the first-phase of pandemic lockdown (6, 7). Our study considered “multimorbidity” as the primary focus, since these individuals have poorer health outcomes and associated with greater healthcare use than single chronic condition (20). We aimed to investigate (1) the association between multimorbidity (by number and type of chronic condition) and routine-care disruptions, (2) how these differ by sociodemographic factors, and (3) the perceived challenges in care management under such situations.

## Materials and Methods

### Study Design, Setting, and Participants

This community-based cross-sectional study was conducted in Khurda district of Odisha, an eastern state of India. There were two reasons for selecting this district: (1) Bhubaneswar, the capital city of the state is located in this district, and thus the first-point of entry for all outside travelers by air. (2) The initial majority of cases were reported from Bhubaneswar city and adjoining rural areas.

As of May 31 2020, the state had reported 2245 cases, including 993 active, 1,245 recovered, and seven fatalities (21). Khurda was one of the most affected districts at that time accounting for one-fifth of cases. Initially, the cases confined to urban areas, spread to rural areas within a span 2 weeks. The district implemented a full-lockdown from the third week of March to the last week of May 2020 (6, 7).

Khurda has a population of around 2.3 million with one municipal corporation (Bhubaneswar) and ten administrative blocks (22). The majority avails health care from either public or private or together. We included both urban and rural community so as to assess context-specific variations if any, across the effect of COVID-19 pandemic on multimorbidity.

### Sample Size and Sampling

Our study being the first to explore multimorbidity management during COVID-19; and given the relevance of timely data, we opted for an exploratory cross-sectional design. The sample size was calculated using Open-Epi software. Drawing from our previous chart-review and with the assumption of 50% of adult (18 years and above age) patients with NCDs having challenges during the pandemic, 95% confidence interval, 80% power, and 1.5 design effect, we arrived at the sample size of 576 (23). To accommodate non-response and incomplete interviews, we rounded up the sample size to 600, and decided to divide it equally between urban (300) and rural (300) settings (24). We adopted a multi-stage clusters random sampling approach to recruit the participants; wherein cluster—a group of population elements constitutes the sampling unit instead of a single individual. The primary reason for such sampling design was the low-resource cost and feasibility, easing out potential data collection challenges at pandemic time.

First, the list of community-dwelling adults (i.e., 18 years or above) having at least one diagnosed chronic disease was obtained from the district health management information system and complemented with data from the local health workers to create the sampling frame. For urban setting, we selected Bhubaneswar municipal corporation (BMC) area while for rural, we took two administrative blocks. From BMC, 10 slums and 10 residential areas were randomly included. From each slum and residential neighborhood, 15 adults were randomly selected thus totaling to 300. For rural areas, six villages were picked up from each administrative block (12 villages) and from each village, 25 adult individuals were recruited randomly (*N* = 300). The number of slums and villages were selected proportionally to population. The detailed-sampling frame is provided in Appendix I (Supplementary Material).

### Data Collection Tool and Technique

The survey was conducted simultaneously in urban and rural settings from 1st to 10th June 2020, the time corresponding to the end of phase I of the lockdown (6, 7). As our respondents belonged to rural, and urban (residential and slum), we adopted a blended approach encompassing both face to face and virtual mode for data collection. For rural participants we preferred personal interview by adhering to all the prevailing COVID-19 norms. For urban and suburbs, given the congestion in the neighborhood, and restrictions of housing societies, presence of containment zone, we adopted telephonic interviews.

Prior approval of the local administration was obtained and fieldwork was done in synchrony with the frontline-health-workers, and local-community or public representatives. All interviews were carried out under the supervision of the research team. Prior to interview, informed consent was obtained and the field team confirmed the presence of chronic disease by checking the prescriptions or medications being consumed. Four trained public health postgraduates with background in nursing, social work and physiotherapy were involved in data collection. We closed data collection once we obtained the required sample size of 300 each in urban and rural area, respectively.

We specifically developed a structured questionnaire—Multimorbidity Assessment Questionnaire during COVID-19 (MAQ-COVID19) for data collection (Appendix II in Supplementary Material). MAQ-COVID19 was adapted from our previously used MAQ-PC (multimorbidity assessment questionnaire for primary care tool (25) and the new constructs were decided by the team comprising psychiatrist, epidemiologist, physician scientist, public health specialist with experience of designing and conducting community as well as primary care based research on multimorbidity.

The MAQ-COVID19 comprised following sections. (1) Socio- demographic characteristics—age, gender, education, income, occupation, and residence. (2) Multimorbidity Assessment—questions on the presence of chronic conditions, duration, treatment status, and routine care. (3) Multimorbidity Management during COVID-19 had three sub-sections: (A) care challenges comprised items on the effect of COVID-19 on routine and emergency care. We used a Likert scale to assess the care challenges with possible answers: “not at all,” “somewhat,” and “a lot” across these dimensions—**D**aily self-care (diet and physical activity), with questions referred to any change during the pandemic in physical, psychological and routine activities. Access to and continuation of prescribed **D**rugs, **D**iagnostics, **D**octor consultation, **D**ay care procedure, **D**ifficulties in treatment seeking. B. changes in disease management-specific behaviors using the Likert Scale, and C. coping or adapting strategies to meet the care needs. (4) Self-rated Health (SRH)—alongside, three questions were on their self-rated physical, mental and overall health during the pandemic. The study referred to the time period from March 15 2020—when the first COVID-19 case appeared in the state—to 31st May 2020 (with a complete lockdown occurring from 3rd week of March to May end 2020) (6, 7).

### Statistical Analysis

The data were entered in Epi info and transferred to Stata 13 (Stata Corp. College Station, TX, USA) for statistical analysis. Frequency (n) and percentage (%) were used for categorical variables and mean for quantitative variables. We used Principal Component Analysis (PCA), dimensionality reduction technique to transform seven co-related variables, which were asked to assess routine care challenges during pandemic in to a smaller number of uncorrelated variables. Here PCA was used to reduce the dimensions of the input features (i.e., care challenges) through projection of eigenvectors of co-variance. These variables include difficulties in **D**aily self-care (diet and physical activity), access to and compliance with prescribed **D**rugs, **D**iagnostics, **D**octor consultation, **D**ay-care procedure and Emergency care. After standardization—all the variables were transformed to the same scale; the covariance matrix was computed. Finally, the eigenvectors and eigenvalues of the covariance matrix were computed to identify the principal components. Factors having eigenvalues more than one were extracted to derive a single variable explaining the “care challenges” during pandemic. We categorized it into a binary variable wherein below or equal median score represents “no significant care challenge” and above median represents “significant care challenge” (26).

We did multivariate logistic regression to analyse the association of socio-demographic characteristics and multimorbidity status with care challenges; odds ratio, adjusted odds, 95% confidence interval for the association and *p*-value for the significance were obtained; *p* < 0.05 were considered significant.

Self-rated physical, mental and overall health was assessed for multimorbidity using binary logistic regression. The outcome variables were self-rated health while presence of multimorbidity was the exposure variable.

### Ethical Considerations

Ethical clearance was obtained from the Institutional Ethical Committee of the ICMR-Regional Medical Research Centre, Bhubaneswar (ICMR-RMRCB/IHEC-2020/027). All participants were briefed on the study objectives and informed consent (telephonic and face-to-face) was obtained. Necessary measures were taken to ensure confidentiality and anonymity of patients.

## Results

We had to approach 720 adults having at least one chronic condition, to obtain our required sample size of 600 (300 rural + 300 urban) with a consent rate of 80%. In rural and urban slum (*N* = 450) interview were face-to-face, while for urban residential areas (*N* = 150) it was telephonic. The socio-demographic characteristic of the respondents is presented in Table 1. The mean age of participants was 55 years with equal proportion of males (49%) and females (51%).

**Table 1:** Socio-demographic characteristics of study participants.

**Demographic characteristics**	**Multimorbidity** **(*N* = 237)** ***n* (%)**	**Non-multimorbidity** **(*N* = 363)** ***n* (%)**	**Total** **(*N* = 600)** ***n* (%)**
**Sex**			
Female	122 (51)	183 (50)	305 (51)
Male	115 (49)	180 (50)	295 (49)
**Age group in years**			
≤30 years	5 (2)	44 (12)	49 (8)
31–45 years	30 (13)	64 (18)	94 (16)
46–60 years	104 (44)	123 (34)	227 (38)
>60 years	98 (41)	132 (36)	230 (38)
**Residence**			
Rural	115 (49)	185 (51)	300 (50)
Urban	122 (51)	178 (49)	300 (50)
**Education**			
No formal schooling	49 (21)	74 (20)	123 (21)
Primary (1st to 7th)	52 (22)	87 (24)	139 (23)
High school (8th to 12th)	58 (24)	98 (27)	156 (26)
Graduation and above	78 (33)	104 (29)	182 (30)
**Occupation**			
Unskilled workers/Farmers	45 (19)	53 (15)	98 (16)
Skilled workers	6 (2)	10 (3)	16 (3)
Business/Public or Private Employee	65 (28)	99 (27)	164 (27)
Homemaker/Student/Retired employee	121 (51)	201 (55)	322 (54)
**Family environment**			
Living alone	6 (3)	10 (3)	16 (3)
Living with spouse/family	231 (97)	353 (97)	584 (97)

Table 2 describes the profile of chronic conditions of the study respondents. Hypertension (44%) was found to be the most-leading condition, followed by diabetes (36%), musculoskeletal morbidities (15%), and acid peptic diseases (9%), respectively. Significant differences (*p* = 0.001) were observed in the prevalence of certain individual chronic conditions between urban and rural participants. Diabetes was higher in urban (43%) than rural (28%) while musculoskeletal morbidities was reported significantly higher (*p* = 0.001) in rural respondents (20%) than urban (10%). Further, the reported prevalence of acid peptic diseases was higher (*p* = 0.001) in rural (14%) than urban (4%) while thyroid disorders were found to be significantly higher (*p* = 0.006) in urban respondents (8%) than their rural counterparts (3%).

**Table 2:** Profile of chronic conditions.

**Major disease conditions**	**Prevalence**			
	**Total** **(*N* = 600)** ***n* (%)**	**Urban** **(*N* = 300)** ***n* (%)**	**Rural** **(*N* = 300)** ***n* (%)**	***P*-value**
Hypertension	261 (44)	127 (42)	134 (45)	0.564
Diabetes	215 (36)	130 (43)	85 (28)	**0.001**
Musculoskeletal disorders	91 (15)	31 (10)	60 (20)	**0.001**
Acid peptic disease	54 (9)	13 (4)	41 (14)	**0.001**
Cancer	46 (8)	23 (8)	23 (8)	1.000
Chronic lung diseases	46 (8)	29 (10)	17 (6)	0.066
Thyroid disorders	31 (5)	23 (8)	8 (3)	**0.006**
Chronic heart disease	28 (5)	19 (6)	9 (3)	0.053
Chronic kidney diseases	23 (4)	13 (4)	10 (3)	0.524
Brain stroke/paralysis	18 (3)	8 (3)	10 (3)	0.632
Psychiatric illness	14 (2)	7 (2)	7 (2)	1.000
Visual impairments	12 (2)	3 (1)	9 (3)	0.142
Hemoglobinopathies	11 (2)	4 (1)	7 (2)	0.545
Disability/deformity	8(1)	6 (2)	2 (1)	0.286
Migraine	5 (1)	4 (1)	1 (0.3)	0.373
Vertigo/hearing impairment	5 (1)	2 (1)	3 (1)	1.000
Epilepsy	4 (1)	2 (1)	2 (1)	1.000
Chronic liver diseases	3 (1)	0 (0)	3 (1)	0.249
Dementia/parkinsonism	1 (0.2)	1 (0.3)	0 (0)	0.317
Hypertension and Diabetes	83 (14)	49 (16)	34 (11)	0.076
Hypertension and Musculoskeletal disorders	23 (4)	12 (4)	11 (4)	0.832
Diabetes and Musculoskeletal disorders	16 (3)	9 (3)	7 (2)	0.612

*Bold values indicates statistically significant*.

The average duration of the chronic conditions was around 9 years, ranging between 1 and 40 years. With regard to normal routine care (prior to COVID-19) for their chronic illnesses, more than 90% had been consulting physicians and taking medications. Approximately, 70% used to follow physical activity/diet restrictions, and 57% were regularly doing laboratory tests (e.g., blood sugar), blood pressure measurement or ancillary investigations.

Around 40% (*n* = 237) of participants were having multimorbidity (two or more chronic conditions) comprising −122 (41%) from urban and 115 (38%) from rural locations. No significant difference was observed in the distribution of multimorbidity across gender, residence, education, occupation and family environment. Multimorbidity was found to be most prevalent in the age group of 46–60 (44%) followed by those of age ≥ 60 years (41%).

Within the group having multimorbidity (*n* = 237), around 73% (*n* = 173) had two conditions, 24% (*n* = 57) had three and very few (*n* = 7) had four chronic conditions. The most frequently reported dyad (combination of two conditions) was “hypertension + diabetes” with a prevalence of 48% (*n* = 83). The second most leading dyad was “hypertension + musculoskeletal morbidities” with 13% (*n* = 23) prevalence followed by “diabetes + musculoskeletal morbidities” having 9% (*n* = 16) prevalence.

Regarding disease specific management behaviors in the past 8 weeks of pandemic restriction, participants reported changes across all dimensions. Around 16% reported changes in physical activity, 12% in diet, 19% had changes in the continuity of treatment, 18% reported deviations in the physician consultation, and 20% reported changes in their routine investigations and health checkups (e.g., blood pressure).

Among study participants, continuation of treatment and routine check-up was notably altered in individuals with multimorbidity (*p* = 0.014) compared to those having single chronic condition (*p* = 0.012) with a strong statistical significance. We further observed a significant difference in daily routine, physical activity, and the continuation of treatment between urban and rural settings during the past 8 weeks. Approximately 26% of respondents residing in urban area had difficulties in their daily routine, while it was 9% for rural patients (*p* = 0.001). Similarly, 23% of urban participants changed their physical activity during the pandemic when compared to 8% of rural participants (*p* = 0.001). Additionally, there was a notable difference in dietary practice change between urban and rural settings (*p* = 0.042), with around 14% of urban changing dietary patterns compared to rural areas (9%).

Concerning the overall effect of COVID-19 on chronic disease care management, individuals with multimorbidity (43%) reported more challenges compared to those with single condition (35%). The most challenging issue was physician consultation (43%) followed by diagnostic investigations (26%). Transport logistics (33%), financial arrangements (26%), mobility-restrictions (21%), and fear of going to hospital owing to the risk of contagion (18%) were prominent reported factors. Those with multimorbidity perceived significantly higher challenges (*p* = 0.004) in physician consultations (62%) compared to single chronic condition (42%). These individuals (54%) also found it more challenging to access hospital services (0.044) than their counterparts with one condition (39%). In urban areas, the respondents experienced significant challenges (*p* = 0.012) in scheduling physician appointments (59%) compared to rural areas (42%).

**Figure 1:**
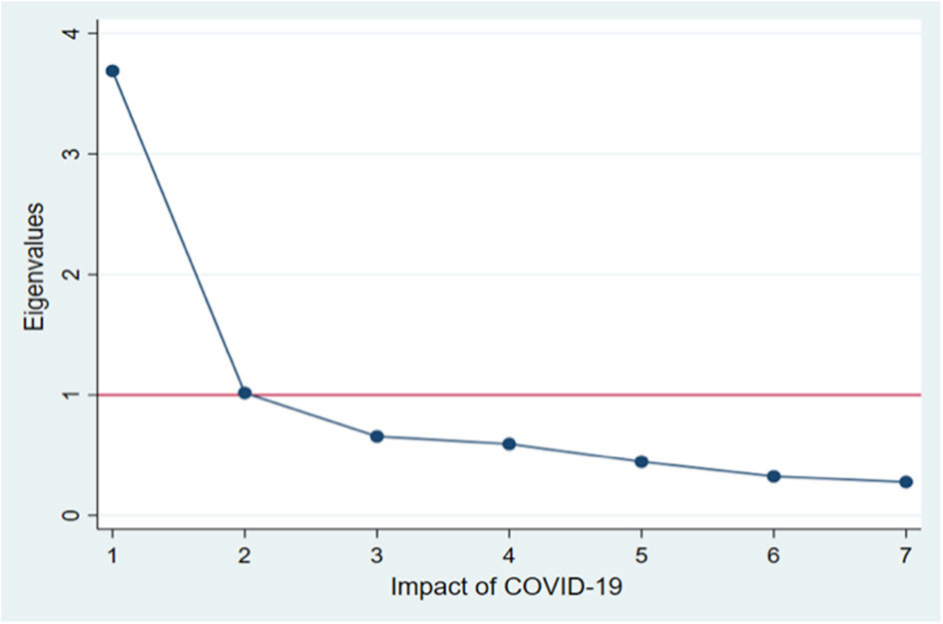
Scree plot of eigenvalues after Principal Component Analysis.

After PCA, out of 600 participants, 229 (38.2%) participants were found to have care challenges more than median value, and remaining 371 (61.8%) were found below or equal to median scores. As per our operational definition, this group was considered to have significant challenges due to COVID-19 Pandemic. An Eigen value for each component was presented in the screeplot (Figure 1). Components with eigen values more than 1 were extracted. First component explains 52% of total variance; when considered first two components, the cumulative variance was 67% (Appendix III in Supplementary Material).

The association of socio-demographic characteristics and morbidity conditions with care challenges during pandemic is presented in Table 3. The female respondents reported greater care challenges than males (45 vs. 32%). The perceived care challenges were significantly higher among younger individuals—less than 45 years (41%), urban dwellers (43%), educated up to graduation and above (46%), retired employee/homemakers (41%), and residing alone or separately from family members (38%). On univariate logistic regression, gender (OR = 1.75), residence (OR = 1.51), and presence of multimorbidity (1.44) were found to be significant factors. However, the multivariate analysis demonstrated that the probability of reporting care challenges was higher twice in female participants than their male counterparts (AOR = 1.96, 95% CI = 1.25–3.1). Having an educational level of graduation or above was more significantly associated with higher care challenges than no formal education (AOR = 1.81, 95% CI = 1.01–3.23). Individuals with multimorbidity were 48% more likely to report significantly higher care challenges than those who had single morbidity (AOR = 1.48, 95% CI = 1.01–2.05).

**Table 3:** Association of socio-demographic characteristics and multimorbidity with care challenges.

**Demographic characteristics**	**No significant challenge in care** **(*N* = 371)** ***n* (%)**	**Significant challenge in care** **(*N* = 229)** ***n* (%)**	**OR [95% CI]**	**AOR [95% CI]**	***p*-value**
**Sex**					
Female	169 (55)	136 (45)	1.75 [1.25, 2.44]	1.96 [1.25, 3.10]	0.003*
Male (Ref.)	202 (68)	93 (32)			
**Age group in years**					
≤ 45 years (Ref.)	85 (59)	58 (41)			
46–60 years	140 (62)	87 (38)	0.91 [0.59, 1.40]	0.90 [0.57, 1.42]	0.654
>60 years	146 (63)	84 (37)	0.84 [0.55, 1.29]	0.85 [0.53, 1.37]	0.511
**Residence**					
Rural (Ref.)	200 (67)	100 (33)			
Urban	171 (57)	129 (43)	1.51 [1.08, 2.10]	1.34 [0.90, 2.02]	0.146
**Education**					
No formal schooling (Ref.)	79 (64)	44 (36)			
Primary (1st to 7th)	92 (66)	47 (34)	0.92 [0.55, 1.53]	0.95 [0.57, 1.61]	0.861
High school (8th to 12th)	102 (65)	54 (35)	0.95 [0.58, 1.56]	0.96 [0.58, 1.70]	0.987
Graduation and above	98 (54)	84 (46)	1.52 [0.95, 2.43]	1.81 [1.01, 3.23]	0.045*
**Occupation**					
Unskilled workers/Farmers (Ref.)	68 (69)	30 (31)			
Skilled workers	10 (63)	6 (37)	1.36 [0.45, 4.08]	0.99 [0.31, 3.13]	0.983
Business/Profession	104 (63)	60 (37)	1.31 [0.77, 2.23]	1.53 [0.79, 2.98]	0.211
Homemaker/Student/Retired employee	189 (59)	133 (41)	1.60 [0.98, 2.59]	1.23 [0.66, 2.28]	0.522
**Family environment**					
Living alone (Ref.)	7 (44)	9 (56)			
Living with spouse/family	364 (62)	220 (38)	0.47 [0.17, 1.28]	0.49 [0.17, 1.39]	0.178
**Morbidity**					
Single (Ref.)	237 (65)	126 (35)			
Multimorbidity	134 (57)	103 (43)	1.44 [1.03, 2.02]	1.48 [1.01, 2.05]	0.043*

Upon additional analysis, we observed a significant association between self-rated physical and mental health with multimorbidity presence. Those with multimorbidity were more likely to report worse physical (OR = 1.49) and mental health (1.65) than having single morbidity. These associations were found to be statistically significant (*p* < 0.05); however, urban respondents self-rated their overall health (70%) as better (*p* = 0.002) than rural (66%). Similarly, mental health (73%) was self- rated better in urban (*p* = 0.011) than rural (62%) (Table 4).

**Table 4:** Association of multimorbidity with self-rated health.

**Multimorbidity**	**Self-rated physical health**		**Self-rated mental health**	
	**Good**	**Bad**	**Good**	**Bad**
Present	143 (60.34)	94 (39.66)	145 (61.18)	92 (38.82)
Absent	252 (69.42)	111 (30.58)	262 (72.18)	101 (27.82)
	OR = 1.49 (1.06, 2.10) *P* = 0.022		OR = 1.65 (1.16, 2.33) *P* = 0.005	

## Discussion

COVID-19 pandemic has posed unique challenges to healthcare delivery with profound implications for patient care especially long-term-illnesses. In this study, we explored the effects of the COVID-19 pandemic on multimorbidity management, and assessed the care spectrum by gender, age, residence, and education through multimorbidity lens to provide greater insights into public health research. Our study sample is reflective of the general population bearing equivalent age and gender distribution with current demographic structure with diverse strata including education and income (27, 28).

We found multimorbidity prevalence to be 40% with no significant difference between urban and rural locations or gender. Across LMICs, the multimorbidity prevalence is reported to be 33.1%, regardless of national gross domestic product (29–31). We had witnessed congruent estimates in our primary care based multimorbidity study with no definitive difference across rural-urban and gender (32).

Multimorbidity was most prevalent in the age group of 46–60 followed by those of age ≥ 60 years. Reports have indicated growing trends of multimorbidity across LMIC with earlier onset of NCDs in the young adult population (31). However, the observed higher magnitude of multimorbidity in a productive age group is a matter of concern. Furthermore, these individuals have to be socially and physically active owing to their functional role and livelihood activities, with potential increased risk of exposure and vulnerability (33). Moreover, especially the working adults with cardio-metabolic (hypertension + diabetes) dyad should stringently adhere to COVID-19 preventive measures, such as physical distancing, wearing mask and hand hygiene (34–36).

Disruptions in routine management for chronic conditions were notably observed in individuals with multimorbidity than those with isolated condition across the care spectrum. Interestingly, we found urban participants reported more deviations in their routine physical activity during the pandemic when compared to rural. The imposed restrictions on mobility and transport could have contributed to this phenomenon in urban population (37). However, the reduced level of physical activity is worrisome as it is necessary for individuals with cardio-metabolic clusters for both glycemic and metabolic health. With a large number of people getting confined to their homes owing to pandemic, the accumulating sedentary lifestyle might aggravate the existing magnitude of NCDs, which further could perpetuate the risk for COVID-19 infection (36). Therefore, it is mandated that individuals need to strike a balance, between physical activity and shelter-at-home norms while exploring home-based physical activity as substitute options.

“Doctor consultation” was the most challenging issue reported among multimorbidity group leading to reduce physical appointments or deferred planned day-care procedures. Urban respondents experienced more challenges in accessing a physician than their rural counterparts. In rural area, public health system through its primary and community health centers, the first-point of care, were providing a range of essential outpatient services during the pandemic time. However, in urban areas, people mostly avail of private health sector. Majority of private clinics were functioning sub-optimally at this period to avert any potential COVID-19 exposure thus disrupting routine patient care services. Moreover, to meet the increasing demands, some private hospitals were made into designated COVID-19 treatment facilities (38).

Accordingly, the governments leveraging on already existing tele-medical system, have started tele-medical oversight by physicians thereby reducing physical visits to facility (39). Mostly clinical decision-making is reasoning based, and telemedicine can offer faster access to subspecialists who may not be immediately available in person. Since healthcare is culturally and contextually embedded, the inclination of patients to avail telecare is important and complementary for physician-patient tele-alliance (40). To have a fuller understanding, it is necessary to explore the physicians' experiences on healthcare provision during pandemic.

Laboratory investigations was next affected care domain. Relocation of laboratory staff from different facilities for COVID-19 testing and the diagnostic procedures requires personal contact and visit to the facility. This was challenging for both the providers and consumers as they had use the personal protective equipment judiciously (41). Majority had no challenges in access to medicine, as many pharmacies were mandatorily kept open during the lockdown.

Our results indicate that within multimorbidity, older adults, living alone in urban area had increased likelihood of disrupted care and could be more vulnerable. Mostly our awareness programs have been directed to prevention and protection against COVID-19. Inclusion of self-care and multimorbidity literacy along with COVID-19 appropriate behavior could reduce the vulnerability and pave path for patient activation (42, 43).

Arranging transport and finance, mobility-restrictions and the fear of contagion at the hospital were the key factors influencing the delay/disruption in seeking care. The restrictive measures have inadvertently added challenges for patients and community. This is inevitable in every pandemic wherein the sole objective of the healthcare system is to combat the spread and reduce the adverse outcomes at the population level. At the same time, given the avidness of COVID-19 for chronic diseases especially multimorbidity, health systems are suggested to have a harmony between COVID-19 prevention measures and NCDs care services.

Multimorbidity is known to incur higher healthcare use, and expenditure with often competing care demands. Previous reports have highlighted the inferior physical and mental health of multimorbidity compared to those with none. In our study, individuals having multimorbidity reported worse physical and mental health than those who had single morbidity (44), and thus could be disproportionately affected by the psychological stress of COVID-19 pandemic (45). This new era of physical-mental multimorbidity further insinuates the importance of integrating mental healthcare with NCD management and COVID-19 measures (46).

As significant number of persons with multimorbidity experienced care challenges, the “one patient—one disease” paradigm tends to lose its relevance and ideally be replaced with “one patient- whole care.” The prevailing clinical practice guidelines for COVID-19 comorbidity management are largely configured around single diseases thus restricting the extrapolation of these protocols to manage multimorbidity (47). A better understanding of individual, family, community and health system level factors underpinning care-pathways would enable development of context-specific patient-centric care protocols.

## Strengths and Limitations

Considering the urgency and time-sensitiveness of the study, we confined to one district, thus possessing restricted scope of generalizability. However, the robustness of our study design, sampling frame and randomness in recruitment of participants have infused substantial scientific rigor and we believe our findings are representative of the broader landscape. Being the first ever to investigate the effect of COVID-19 pandemic on multimorbidity management in any LMICs, community setting, scheduling of the study immediately after the first-phase of lockdown, high participant response rate and using a specifically developed and validated questionnaire for data collection are our major strengths.

## Future Implication and Conclusion

Multimorbidity significantly impacts both routine and emergency care vis-à-vis single chronic morbidity, the challenges being more pronounced in urban dwellers, older adults, and living alone. Telehealth could be considered as a complementary approach since it is well-suited for situations wherein infrastructure is intact and physicians are available to attend to patients.

The continuation of preventive and management services of NCDs in tandem with COVID-19 containment measures should additionally include self-care and multimorbidity literacy toward patient activation. The observed higher presence of multimorbidity in younger population requires tailored health advisories harnessing on digital and traditional communication with stringent compliance to COVID-19 protection measures. The region-specific pandemic healthcare preparedness plan must strategically incorporate measures to reduce risk of infection and outcomes while resuming economic-activity. Patient-centric-protocols to manage commonly occurring multimorbidity clusters is a therapeutic necessity.

Researchers in LMICs may undertake similar studies to assess care challenges toward managing multiple chronic conditions or multimorbidity using MAQ-COVID-19 questionnaire in their settings. Since “physician consultation” emerged as a major perceived challenge, future studies should explore the challenges of healthcare providers to garner an in-depth understanding. Furthermore, qualitative inquiry into care management, care experience and needs of the urban-dwelling older adults living alone could provide key insights to the public health planners.

## Data Availability Statement

The raw data supporting the conclusions is with the corresponding author and can be made available on reasonable request and prior approval.

## Ethics Statement

The studies involving human participants were reviewed and approved by ICMR-Regional Medical Research Centre, Bhubaneswar (ICMR-RMRCB/IHEC-2020/027) State Ethical Review Committee, Department of Health and Family Welfare, Odisha Government (Letter No. 13260/MS). The patients/participants provided their written informed consent to participate in this study.

## Author Contributions

SP conceptualized the study. SP, KS, SK, and PM designed the study methodology and developed the tool. KS, SK, and PM were involved in data collection. SK, KS, and AU analyzed the data. KS and SK prepared the first draft of the manuscript which was then refined and finalized by PM and SP. The manuscript has been read and approved by all the authors.

## Conflict of Interest

The authors declare that the research was conducted in the absence of any commercial or financial relationships that could be construed as a potential conflict of interest.

## Abbreviations

AOR: Adjusted odds ratio
APD: Acid peptic disease
APL: Above poverty line
BPL: Below poverty line
PHC: Primary health center
CHC: Community health center
CI: Confidence interval
IRR: Incidence rate ratio
LMIC: Low and middle income countries
NCD: Non communicable disease
SRH: Self-reported Health
CVD: Cardiovascular disease
HTN: Hypertension
COPD: Chronic obstructive pulmonary disease
CKD: Chronic kidney disease
AKI: Acute kidney injury
MAQ: Multimorbidity Assessment Questionnaire
WHO: world health organization.
