# Supplementary material for: Managing Multimorbidity (Multiple Chronic Diseases) Amid COVID-19 Pandemic: A Community Based Study From Odisha, India
Source: Front Public Health. 2021 Feb 1;8:584408. doi: 10.3389/fpubh.2020.584408 (PMC7882709; doi:10.3389/fpubh.2020.584408)
Supplement: Supplementary file 1 [file Data_Sheet_1.PDF]

MAQ COVID-19

Multimorbidity Assessment Questionnaire during COVID-19 Pandemic

Date of data collection: **DD / MM/YY** **Block Code:** **District:** **State:**

1. Socio-demographic Information

<table>
<tr><td>Participant ID:</td><td colspan="2">Age (in Years):</td></tr>
<tr><td>Household ID :</td><td colspan="2">Highest years of completed education (in years):</td></tr>
<tr><td>Religion: a. Hindu b. Islam c. Christian d. Others</td><td colspan="2">Ethnicity : a. Tribal b. Non-tribal</td></tr>
<tr><td>Social Security Card: a. Yes b. No</td><td colspan="2">Gross family expenditure per month (in INR):</td></tr>
<tr><td>Total number of family members:</td><td colspan="2">Marital Status:
a. Married b. Separated c. Not Married d. Widowed</td></tr>
<tr><td>Family environment:
a) Living alone
b) Living with spouse
c) Living with children/other family members</td><td>Gender:
a) Male
b) Female
c) TG
d) Other</td><td>Present place of living:
a) Urban (residential)
b) Urban slum
c) Rural
d) Suburban/ Peri Urban</td></tr>
<tr><td>Current Occupation:
a. Paid Work b. Self employed
c. Non-paid work such as volunteer d. Student
e. Home Maker f. Unemployed (Health ground)
g. Retired h. Unemployed (Other reasons)
i. Others (Specify………….)</td><td>Current occupation Since:
…………years

Monthly expenditure on health care : …………INR</td><td>Do you have any health insurance?

Yes – Public Scheme
Yes- private
No health insurance</td></tr>
</table>

2. Multimorbidity Assessment: Could you please tell us about each of your long-term illness (Please refer to the list)

Sl . No.	Long standing Condition	Duration in Years	Consulting doctor	Taking treatment	Doing any physical activity diet restriction	Any supportive care	Regular lab tests	Any day care procedure

**List of Chronic Conditions**	**Have you ever been diagnosed with any of the following long term or long standing health conditions ?**				
Diabetes (1)	Hypertension/ High Blood Pressure (2)	Respiratory Diseases (COPD/ Asthma) (3)	Acid Peptic Disease (4)	Thyroid disorders (5)	Chronic skin disease (6)
Chronic Backache (7)	Arthritis (8) Osteoarthritis / RA	Psychiatric illness (9) Mention type	Visual impairments (retina/ cataract/ glaucoma) (10)	Piles / constipation/ Irritable Bowel Syndrome (11)	Chronic non-healing wound (12)
Hearing Impairment (13)	Migraine (14)	Chronic liver disease or pancreatitis (15)	Hemoglobinopathy / Anaemia) (16)	Chronic Kidney Disease (CKD) (17)	Filariasis (18)
Chronic Heart disease (19) (Ischemic heart disease/ Heart failure)	(20) Dementia Parkinsonism	Alcohol & substance abuse (21)	TB (22)	Cancer - mention type (23)	Disability/ deformity (24)
Brain stroke/ paralysis (25)	Epilepsy (26)	HIV (27)	Vertigo (28)	Obesity (29)	Other _____

© Sanghamitra Pati, drsanghamitra12@gmail.com

3. Effect of COVID-19 Pandemic on Multimorbidity Management

A. Routine Care for Chronic Illness					
**How much COVID-19 Pandemic has affected any of your routine long-term illness care**	Not at all/ No change	Little	Somewhat	Quite a bit	A Lot
Has your daily routine been affected?					
Has there been a change in physical activity?					
Has there been a change in diet?					
Has there been any discontinuation in treatment for your chronic disease					
Has there been any change in doctor consultation					
Has there been any change in your routine blood tests or BP check or other investigations?					
**B. Chronic Illness Specific Health Care**					
**How much COVID-19 pandemic has affected any of these in your chronic illness management in the past 8 weeks?**	Not at all	Little	Somewhat	Quite a bit	A Lot
Did you ever feel the need to consult a doctor in the past 8 weeks?					
Did you feel the need to visit a hospital or clinic?					
Did you face difficulty in getting a doctor appointment for your chronic disease					
Did you experience any difficulty in getting medicine?					
Did you feel any difficulty in getting any investigations done for your chronic disease					
Did you feel any difficulty in getting any day care procedures like chemo, dialysis, physiotherapy for your chronic disease					
Did you feel difficulty in getting any emergency care for this?					
Did you feel difficulty in reaching the hospital / clinic/ day care					
Did you experience any delay in seeking care?					

© Sanghamitra Pati, drsanghamitra12@gmail.com

**In the past eight weeks which one of the following was the most challenging for you in getting access to?**	Not at all	Little	Somewhat	Quite a bit	A lot
Doctor consultation					
Drugs or medicines					
Laboratory Tests/ Diagnostics / Investigations done					
Day care / Dialysis					
Emergency care					
Visiting the clinic /hospital					
**Could you please tell us if any of the following issues led to disruption of your healthcare during COVID-19 pandemic and if so, how much.**					
**How much any of these troubled you in the past 8 weeks ?**	Not at all	Little	Somewhat	Quite a bit	A lot
Getting money					
Arranging Transportations					
Getting a person to accompany					
Support from family					
Mobility Restriction					
Lock down					
Fear of going to hospital					
Dint know whom to contact					
Social Restriction					
Any other – Please specify					
**How much support you got from the following in the past 8 weeks?**	Not at all	Little	Somewhat	Quite a bit	A lot
Family					
Friend					
Neighbour/ Neighbourhood					
Informal Care support					
Tenant					
Health worker					
Health centre					
Government helpline					
Local administration					
Police					
Community leader					
NGO /Social worker/Club					
Media					
Any other – Please specify					
C. **Managing through chronic illness care needs**					
**How did you manage your above care needs in the past 8 weeks?**	Not at all	Little	Somewhat	Quite a bit	A Lot
How difficult it was for you to manage the care needs					

© Sanghamitra Pati, drsanghamitra12@gmail.com

4. Self-Rated Health

How would you rate your health in the past eight weeks?	Excellent	Very good	Good	Fair	Poor
In general, how would you rate your overall health					
In general, how would you rate your physical health					
In general, how would you rate your mental health					

Compared to a year ago, how would you rate your health in general now?

Much better than a year ago		Somewhat worse than a year ago	
Somewhat better than one year ago		Much worse than a year ago	
About the same as a year ago			

Think about a scale of 0 to 100, with zero being the least desirable state of health that you could imagine and 100 being perfect health. What number, from 0 to 100 would you give to the state of your health. On average, over the last 3 days?

0	10	20	30	40	50	60	70	80	90	100

© Sanghamitra Pati, drsanghamitra12@gmail.com
