# Supplementary figures and images for: Managing Multimorbidity (Multiple Chronic Diseases) Amid COVID-19 Pandemic: A Community Based Study From Odisha, India
Source: Front Public Health. 2021 Feb 1;8:584408. doi: 10.3389/fpubh.2020.584408 (PMC7882709; doi:10.3389/fpubh.2020.584408)

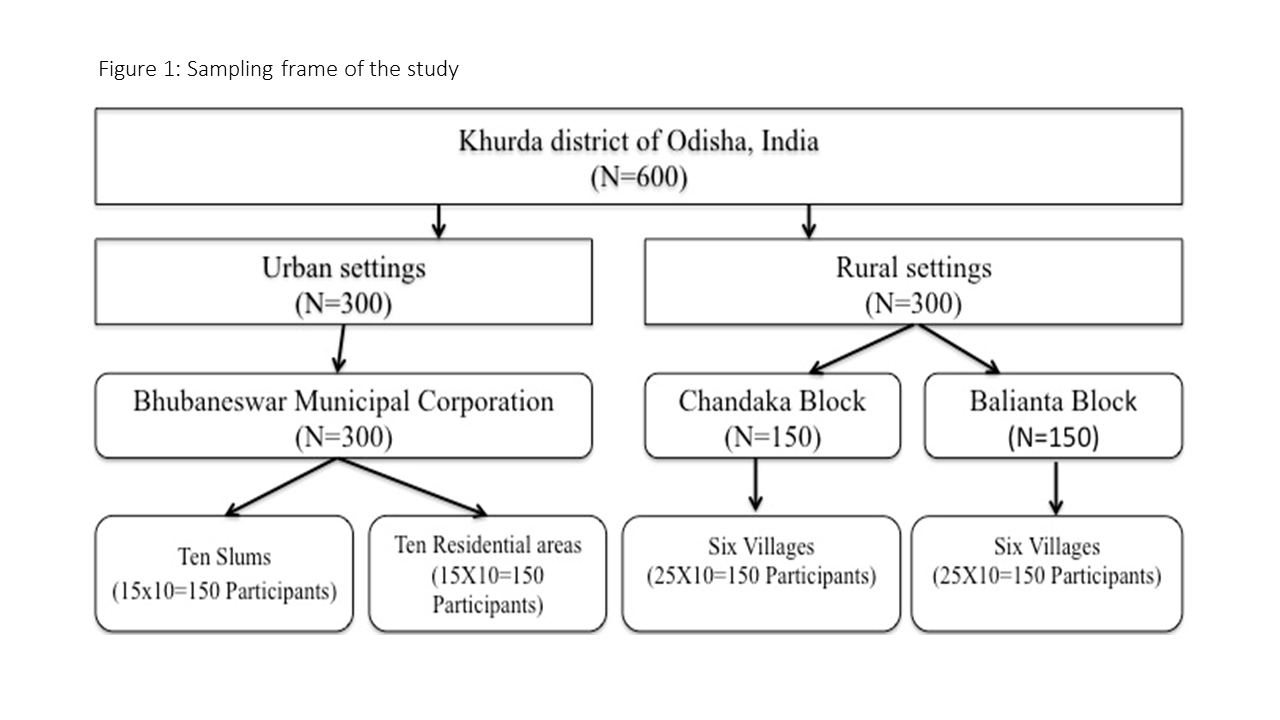

Supplement: Supplementary file 2 [file Image_1.TIF]
