# Supplementary material for: Managing Multimorbidity (Multiple Chronic Diseases) Amid COVID-19 Pandemic: A Community Based Study From Odisha, India
Source: Front Public Health. 2021 Feb 1;8:584408. doi: 10.3389/fpubh.2020.584408 (PMC7882709; doi:10.3389/fpubh.2020.584408)
Supplement: Supplementary file 3 [file Table_1.docx]

Appendix III:

Table 5: Total variance explained by each component

| Component | Initial Eigenvalues | | | Extraction Sums of Squared Loadings | | |
| --- | --- | --- | --- | --- | --- | --- |
|  | Total | % of Variance | Cumulative % | Total | % of Variance | Cumulative % |
| 1 | 3.689 | 52.695 | 52.695 | 3.689 | 52.695 | 52.695 |
| 2 | 1.017 | 14.523 | 67.218 | 1.017 | 14.523 | 67.218 |
| 3 | .656 | 9.378 | 76.596 |  |  |  |
| 4 | .594 | 8.479 | 85.075 |  |  |  |
| 5 | .445 | 6.360 | 91.434 |  |  |  |
| 6 | .323 | 4.616 | 96.051 |  |  |  |
| 7 | .276 | 3.949 | 100.000 |  |  |  |
